# Thermal Damage Estimate Artifact Following Antecedent Biopsy: A Case Report

**DOI:** 10.7759/cureus.31913

**Published:** 2022-11-26

**Authors:** Salma M Bakr, Pranish A Kantak, Med Jimson D Jimenez, Hailey C Budnick, Jeffrey Raskin

**Affiliations:** 1 Department of Neurosurgery, Faculty of Medicine, Ain Shams University, Cairo, EGY; 2 Department of Neurosurgery, Henry Ford Health System, Detroit, USA; 3 Department of Neurosurgery, Rosalind Franklin University of Medicine and Science, North Chicago, USA; 4 Department of Neurosurgery, Indiana University School of Medicine, Indianapolis, USA; 5 Department of Neurosurgery, Northwestern University Feinberg School of Medicine, Chicago, USA; 6 Department of Pediatric Neurosurgery, Ann & Robert H. Lurie Children’s Hospital of Chicago, Chicago, USA

**Keywords:** laser interstitial thermal therapy, epilepsy, laser artifact, litt, mrglitt

## Abstract

MR-guided laser interstitial therapy (MRgLITT) is becoming more commonly used for minimal access approaches to intracranial lesions of all etiologies. The short-term safety profile of MRgLITT is favorable compared with sweeping incisions and open craniotomies, especially for lesions located in deep, periventricular, and highly eloquent areas. The Visualase software (Medtronic Inc., Minneapolis, MN, USA) has multiple adaptations to assist with this safety margin, including the thermal damage estimate (TDE), which applies predictive mathematical modeling to a two-dimensional (2D) graphical representation. TDE has been shown to highly correlate with actual tissue destruction in a priori MRgLITT cases and to anecdotally be imprecise when MRgLITT is combined with biopsy. We present a case regarding a 17-year-old male patient with intractable focal epilepsy. He underwent stereotactic biopsy and then ablation where it was shown that TDE is ~35% larger in the coronal plane than in the actual ablation zone. Air may have caused this artifact in the biopsy cavity, which affected the proton resonance frequency (PRF) and caused TDE pigment deposition. We believe in the need for a more comprehensive understanding and investigation regarding this TDE artifact. Future prospective studies into MRgLITT should attend carefully in cases where it is combined with biopsy.

## Introduction

Laser ablation therapy is a minimal access procedure that has proved its efficacy and wide range of applicability in recent years. MR-guided laser interstitial therapy (MRgLITT) has been successfully applied to numerous intracranial pathologies, such as primary and metastatic tumors, epilepsy, radiation necrosis, and chronic pain [1-9]. MRgLITT was shown effective in treating hypothalamic hamartoma patients with 93% free of gelastic seizures at the one-year mark postoperatively [6]. The MR compatibility of laser ablation systems has provided an avenue to monitor brain parenchymal temperature changes within and adjacent to the lesion through MR thermometry [10]. With this technology, an area of irreversible thermal damage is estimated, and the operating surgeon is provided with a near real-time image of the lesioning process [11]. This ability provides a clear advantage when compared to other ablation techniques such as radiofrequency, which treats a thermal dose without the ability for monitoring [5,11]. In this case, a needle biopsy was performed prior to laser ablation.

The Visualase system (Medtronic Inc., Minneapolis, MN, USA) is an MR-guided laser ablation system consisting of a 15 W, 980-nm diode laser generator, which powers a fiber-optic probe with a light-diffusing tip of either 3 mm or 10 mm, surrounded by a 1.65-mm diameter outer cooling catheter. This system is coupled with an image-processing workstation that interfaces with the MRI [5,11]. The Visualase workstation provides various software specifications that enhance the safety of thermal ablation. The surgeon can choose low-temperature limit target points on anatomical background images around the periphery of a lesion to protect adjacent structures; exceeding the low threshold temperatures prompts an automatic system shutoff with the cessation of laser delivery [5,11]. The high-temperature limit is applied near the laser tip and switches off the laser when the high threshold is reached. In addition to the selection of target points, the system also calculates real-time thermal damage estimates (TDE) based on the MRI pixel shift of the target tissue in response to thermal damage [12]. The TDE appears as an orange overlay on an anatomical reference MRI scan on the Visualase workstation. These safety features have been crucial in allowing neurosurgeons to strike a balance between under-ablation, resulting in suboptimal treatment or treatment failure, and over-ablation, resulting in carbonization, charring, and vaporization of surrounding tissues [5,13,14]. There have not been studies understanding how these safety features change when performing an antecedent biopsy, which involves aspirating the target tissue to obtain a histological sample immediately before thermoablation.

Unfortunately, as with any computerized imaging modality, there is a risk of inaccuracy and error. To our knowledge, this has been scarcely reported in the literature in the setting of intracranial MRgLITT procedures. There is a previous study showing real-time MR-guided laser thermal therapy to have a specificity of 98% and a sensitivity of 79% in the ablation of necrotic animal tissue [15]. We report a case highlighting the limitations of TDE in determining the extent of ablation following antecedent biopsy.

## Case presentation

A 17-year-old male patient with intractable focal epilepsy was evaluated by the comprehensive epilepsy program, and the consensus opinion was that his left parietal tumor was causative. He underwent a stereotactic biopsy of the lesion followed by MRgLITT in the same session.

**Figure 1:**
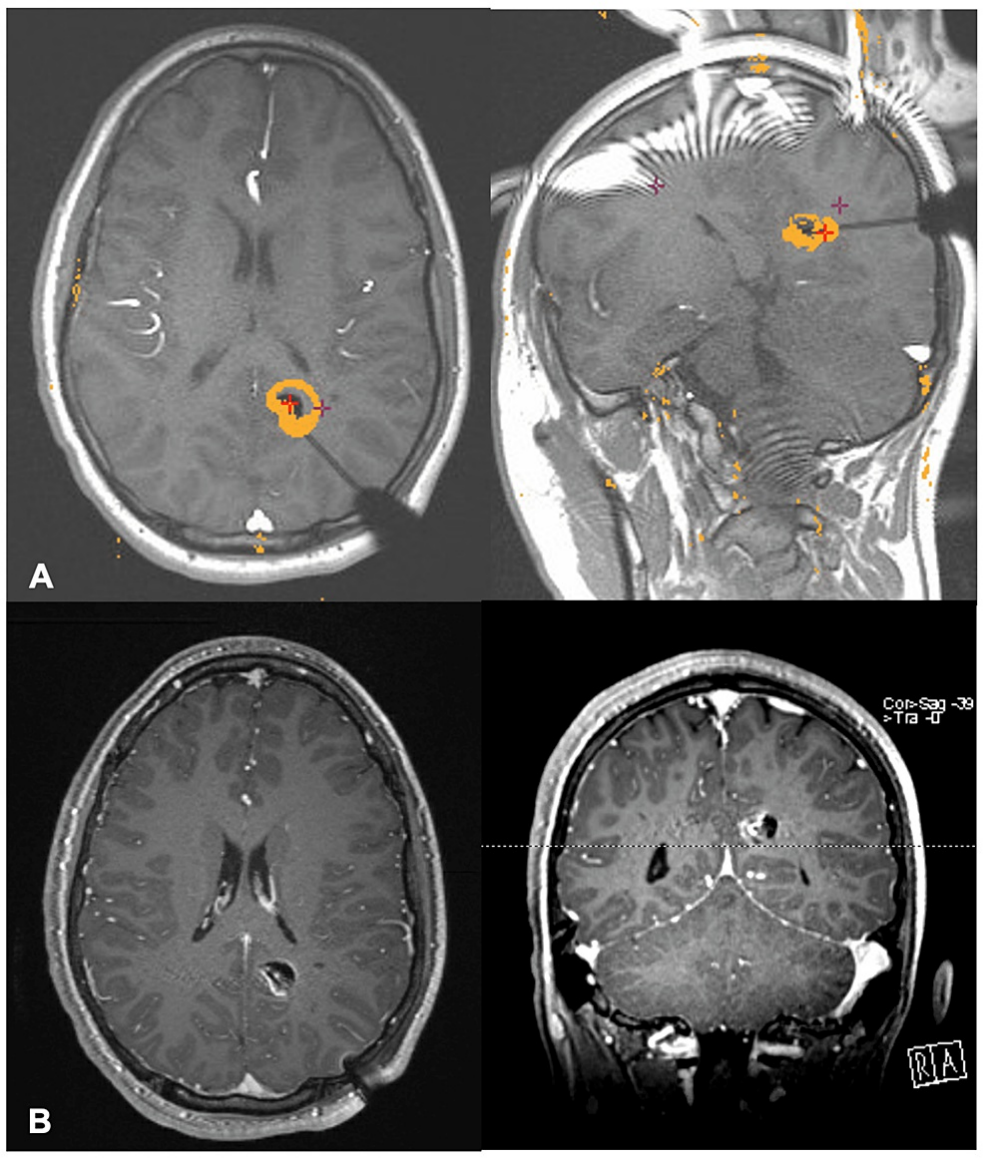
TDE Ablation Estimate Measurements A: TDE T1 sequences showing ablation estimate measurements of 21.9 mm in the axial and 21.2 mm in the coronal oblique planes. B: Immediate post-ablative T1 post-contrast sequences showing true ablation measurements of 15.1 mm in the axial and 13.7 mm in the coronal planes. TDE: thermal damage estimate

**Figure 2:**
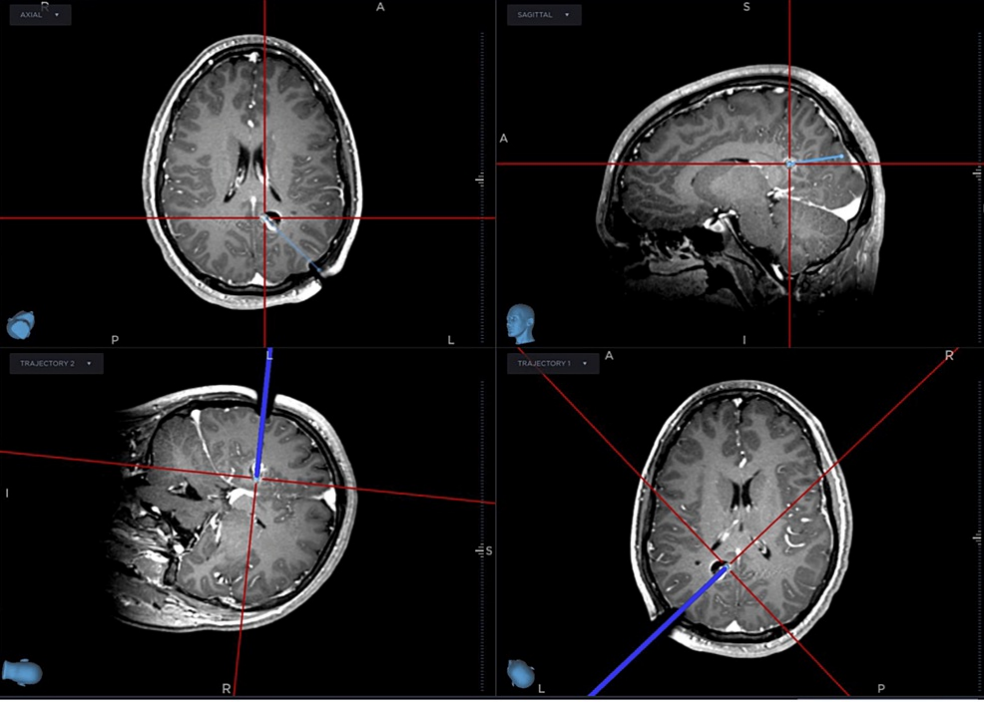
T1 Post-Contrast MRI Following Surgery From top left clockwise: Axial, sagittal, and two orthogonal trajectory views are shown extending into the ablation cavity on this T1 post-contrast MRI following surgery. The cavity measures 14.3 × 11.5 × 14.2 mm in the axial plane. MRI: magnetic resonance imaging

Cranial fixation was achieved using a Cosman-Roberts-Wells (CRW) CT head ring. He was positioned, prepped, and draped in the usual fashion. Coordinates obtained from the Medtronic Stealth system were used to set the CRW precision arc, and these coordinates were checked using the phantom base prior to securing the precision arc to the head ring. A left occipital trajectory was chosen, the scalp was infiltrated with Lidocaine and incised, the bone was drilled, and the dura was palpated and opened. A 2.1-mm reducing tube was secured in the CRW stage through which the biopsy needle was advanced to the target. An O-arm spin with a needle to depth was co-registered to the preoperative plan, confirming that the needle tip was within the lesional target. The lesion was then biopsied and sent to pathology. A needle biopsy was performed with saline in line for aspiration and drizzled down the open cannula to minimize air accumulation at the target. The needle was removed, and the wrench guide was used to place a 25-mm PMT anchor bolt. The distance to the target was measured, the Visualase cooled laser applicator system (VCLAS) was inserted to adequate length, and a 10-mm laser was secured in situ. The patient was cautiously transported to a diagnostic Siemens MRI suite. The applicator lines were passed through a wall port; a volumetric T1 sequence was acquired. Axial and coronal plane T1 sequences were used as anatomical backgrounds for TDE and MR thermometry. Immediately before laser activation, the phase reference was reset. A test dose was initially applied at 4.65 W for 19 seconds to warrant adequate location, followed by a 7.50 W dose for 140 seconds, for which the TDE and MR thermometry are shown (Figure 1A). Very rapidly, the TDE enlarged to a maximum extent of 21.9 mm in the axial plane and 21.2 mm in the coronal plane. T1 post-contrast demonstrated 15.1 mm in the axial plane and 13.7 mm in the coronal plane (Figure 1B). The extent of ablation was thought to be sufficient and at least 100%, the child was transported back to the operating room where hardware was removed, and a single stitch was placed to close the wound. The patient was observed in the recovery room and discharged one day after surgery and remains seizure-free at six months. Figure 2 shows T1 post-contrast MRI following surgery where the extent of the ablation was measured to be 12 mm^3^.

## Discussion

The incorporation of MR thermometry and TDE has rejuvenated the interest of neurosurgeons in MRgLITT and has catapulted this thermal delivery technology above radiofrequency ablation. With near real-time thermal imaging and instant ablation feedback, along with the ability to treat lesions with otherwise high corridor-associated morbidity, this system has tremendous potential for patient care [13,14]. The temperature of each pixel is calculated using the shift in proton resonance frequency (PRF), which is due to the change in tissue characteristics in response to thermal therapy observed on gradient-recalled echo (GRE) imaging sequences [10,12,16-19]. The history of change in each pixel temperature and corresponding time are employed in the Arrhenius rate process model equation, which calculates an estimate of irreversible tissue damage, and plotted on a two-dimensional (2D) image as an orange TDE overlay on the Visualase workstation computer [10,11,16,20,21]. These thermal imaging metrics are repeatedly acquired and analyzed about every five seconds, resulting in a near real-time update of the extent of ablation [22]. 

Unlike temperature maps visualized during MR thermometry, the Arrhenius model takes into consideration the cumulative effect of repetitive heat delivery to the tissue, regardless of intervening temperature breaks or laser cannula retraction during the ablation process, demonstrating a more accurate end-lesion estimate [13]. The correlation between TDE and actual tissue destruction has been assessed by McNichols et al. [13] in canine brains by comparing the TDE to the histopathological and gross pathological changes of ablated tissues and lesion boundaries in the specimens, reporting no significant differences between assessment modalities. Carpentier et al. reported the first experience in applying the TDE in association with MRgLITT for metastatic focal brain tumors in human cases [11], reporting remarkable similarity between TDE and the post-ablative MRI. The authors, however, described their avoidance of performing tumor biopsies to circumvent any possible risks of hemorrhage or the introduction of air bubbles into the lesion, which would create artifacts during MR thermometry. In a series of 20 patients treated with MRgLITT, Jethwa et al. [5] reported extreme accuracy of TDE while also mentioning several causes for inaccuracy, including the introduction of steam or blood into the lesion bed to be ablated or possible movement of a patient resulting in artifact formation [5]. Most recently, a study by Patel et al. [21] has focused explicitly on evaluating the TDE as a measure of the extent of ablation. In this study, 17 patients with brain tumors and five patients with focal epilepsy underwent MRgLITT, and two raters examined the differences between the area of ablation on MRI and the area of TDE both reporting no significant differences [21]. The authors further discussed the limitations of TDE, which, as with any other imaging modality, can never be expected to be error-free. The lack of direct visualization of the tissue and histopathological analysis renders TDE merely predictive and not to be taken as a precise measure of ablation.

Lesion morphology presents another concern to the authors, given that their evaluation that was based on spherical lesions that demonstrated a rather uniform distribution of thermal variation in shape from an ellipsoid could result in a lack of generalizability of their conclusions [21]. The authors emphasize how the 2D model of TDE is a significant limitation, as opposed to a 3D model that would demonstrate the lesion and the ablation process more accurately [21]. Early in the advent of medical laser applications, one of the main challenges was creating a laser that would work on various tissues. Tissue-dependent variations in the scattering properties of light can significantly change local thermodynamics [23,24]. MRgLITT of brain tumors requires precise anatomical planning, although a lack of understanding of local thermodynamics based on the tissue composition may limit a maximal ablation [25,26]. Finally, all suspected tumors should have histological confirmation prior to surgery because biology sometimes affects the extent of surgery. If the histology of a lesion is unknown before ablation, antecedent biopsy immediately beforehand and during the same surgery can affect the thermography.

## Conclusions

In our study, we observed that the ablated tissue was far less than predicted by the TDE. As shown in Figure 1, the TDE was 21.9 mm in the axial plane and 21.2 mm in the coronal oblique plane, whereas the measurement of actual ablated tissue on immediate postoperative contrasted T1 MRI images was 15.1 mm in the axial plane and 13.7 mm in the coronal plane. This discrepancy between predicted and actual ablation has been observed colloquially but not commonly published. As described by a previous study, performing biopsies prior to laser ablation, such as in our case, can cause artifacts and therefore inaccurate TDE results. Our case study reveals a TDE 31% and 35% larger than actual ablation in the axial and coronal planes, respectively. We believe that this is caused by air in the biopsy cavity, seen in Figure 1B, which affects the PRF and causes deposition of TDE pigment.

Furthermore, this case proceeded extremely quickly following laser activation, and the phase reference had been immediately reset, so this was not a contributor to the inaccurate TDE. Figure 2 demonstrates post-contrast MRI following surgery. Given the very close approximation of the TDE to the actual ablated tissue without antecedent biopsy, future prospective studies into MRgLITT should attend carefully to such a mismatch in a similar surgical paradigm.

MRgLITT is an effective method of treating intracranial lesions with or without antecedent biopsy. The TDE is commonly accurate in the prediction of ablated tissue, although when paired with antecedent biopsy, this can be inaccurate. Our case demonstrates that this inaccuracy can be as high as 35% of the predicted volume and may lead to under-ablation and treatment failure.
